# The influence of interval training combined with occlusion and cooling on lipid profile in young healthy people

**DOI:** 10.3389/fphys.2026.1858294

**Published:** 2026-07-27

**Authors:** Bartłomiej Ptaszek, Szymon Podsiadło, Rafał Niżankowski, Piotr Mika, Ewa Sadowska-Krępa

**Affiliations:** 1 Institute of Applied Sciences, University of Physical Culture in Krakow, Krakow, Poland; 2 Institute of Clinical Rehabilitation, University of Physical Culture in Krakow, Krakow, Poland; 3 Sano Science, Centre for Computational Medicine, Krakow, Poland; 4 Institute of Sport Sciences, Academy of Physical Education in Katowice, Katowice, Poland

**Keywords:** BFR, exercise intervention, HIIT, lipid profile, Vasper

## Abstract

**Introduction:**

There is growing evidence supporting the use of interval training and/or low-impact blood flow restriction exercises for musculoskeletal rehabilitation. The aim of this study was to evaluate the effect of interval training combined with occlusion and cooling on lipid profile changes.

**Materials and methods:**

The study included 30 young, healthy, and untrained people. The Vasper training system was used—high intensity interval training with the simultaneous use of occlusion and local cryotherapy. Blood samples were collected from the participants six times (2 weeks before the start of training, on the day of training, after the first training session, after the 10th training session, after the 20th training session, and 2 weeks after the end of training). The subjects were randomly divided into three groups: exercises only (controlled), occlusion, and occlusion with local cryotherapy.

**Results:**

Analysis of the results showed changes in the mean HDL values in the OCC-COO (F = 3.6868, p = 0.007744, Eta-squared = 0.315467) and OCC (F = 3.1597, p = 0.020789, Eta-squared = 0.344959) groups. Training in both groups resulted in a decrease in HDL levels, followed by stabilization.

**Conclusions:**

HIIT with occlusion and cooling, or occlusion alone, may negatively impact HDL levels in young, healthy individuals. Regular, submaximal interval exercise without additional modifications appears to be safe and has no negative impact on lipid profiles. Changes in HDL levels are observed after a series of training sessions, not after a single training session.

## 1 Introduction

High-intensity interval training (HIIT) is characterized by repeated bouts of short-duration exercise (ranging from <60 s to approximately 8 min) performed at vigorous (70%–90% of maximal heart rate [MHR] or 14–16 on the Borg rating of perceived exertion [RPE] scale) to near-maximal intensities (≥90% MHR or ≥17 RPE), interspersed with periods of active (40%–70% MHR or RPE 8–13) or passive recovery lasting 1 min–5 min. Originally introduced in the mid-20th century to enhance athletic performance, HIIT has since evolved, with contemporary protocols designed for non-athletic populations emphasizing reduced time commitment while eliciting substantial physiological and psychological adaptations compared with moderate-intensity continuous training (MICT) (Norton et al., 2010; Seiler and Intervals, 2019). The Vasper training system (Vasper Systems, USA) is an innovative approach to exercise aimed at improving fitness and overall health. It combines elements of compression, cold therapy, and interval training. The term ‘Vasper’ stands for Vascular Performance, emphasizing its main focus on optimizing efficacy of blood vessels. The essence of the system is the use of specialized equipment, including the Vasper device and cooling cuffs. The Vasper is essentially an exercise bike or elliptical trainer equipped with pneumatic compression technology. One of the primary advantages of the Vasper training system is its effect on cardiovascular function.

Metabolic disturbances—characterized by elevated plasma concentrations of low-density lipoprotein cholesterol (LDL-C), triacylglycerols (TAG), and total cholesterol (TC), alongside reduced levels of high-density lipoprotein cholesterol (HDL-C)—are well-established risk factors for the development of cardiovascular diseases (CVD) (Soares-Miranda et al., 2012; Rodríguez-Colon et al., 2015; Rosenson et al., 2016). Among modifiable lifestyle factors, insufficient physical activity and excessive caloric intake play a critical role in the etiology of metabolic disorders (Tran and Zimmerman, 2015) and CVD, which together remain leading causes of global mortality (Pedersen, 2009; Kaess et al., 2015).

HIIT has been extensively investigated across a wide range of populations and settings. In healthy individuals, including children and adolescents, it has demonstrated beneficial effects on cardiovascular fitness, body composition, and metabolic health, both in controlled laboratory conditions and real-world environments such as school-based physical education programs (Costigan et al., 2016). In adult and older populations, HIIT has been shown to improve cardiovascular function, muscular strength, and overall functional capacity in both recreational and clinical contexts (Hwang et al., 2016). Moreover, HIIT has been widely implemented in clinical populations, including individuals with obesity, metabolic syndrome, cardiovascular diseases, and cancer survivors, resulting in significant improvements in various health-related outcomes (Weston et al., 2014; Jaureguizar et al., 2016; Adams et al., 2017; Ellingsen et al., 2017; Jurio-Iriarte and Maldonado-Martín, 2019). Notably, current evidence suggests that HIIT can be safely performed by high-risk groups—such as patients with cancer, metabolic disorders, hypertension, and coronary artery disease (CAD)—and may contribute to sustained improvements in cardiovascular health. In addition, HIIT is commonly employed in athletic populations, where it effectively enhances key performance parameters, including aerobic capacity, anaerobic power, endurance, and recovery efficiency (Laursen and Jenkins, 2002). The combination of compression and interval training improves blood flow and stimulates the circulatory system (Anz et al., 2018; Callanan et al., 2021). Vasper training also significantly increases nitric oxide production, leading to improved blood flow and lower blood pressure while reducing the risk of cardiovascular diseases such as hypertension and atherosclerosis (Boeno et al., 2018). Moreover, Vasper cooling technology plays a vital role in enhancing exercise performance and post-workout recovery. Despite the growing body of literature supporting the beneficial effects of HIIT on cardiovascular and metabolic outcomes, findings remain heterogeneous. While some studies have reported significant improvements in maximal oxygen uptake (VO_2_max), blood pressure, and lipid profiles (Weston et al., 2014), others have demonstrated limited or no observable effects (Berglund et al., 2021).

Although numerous studies have examined the impact of different exercise modalities on lipid profiles, the effects of combining HIIT with occlusion and cooling strategies remain unclear. Therefore, the aim of the present study was to investigate the effects of a HIIT program combined with occlusion, as well as occlusion with concurrent cooling, on triglyceride concentrations and cholesterol fractions. We hypothesized that combining HIIT with blood flow restriction and cooling would result in more favorable changes in the lipid profile than HIIT alone.

## 2 Materials and methods

The research was conducted at the University of Physical Culture in Krakow. The participants were students who were enrolled in the study after undergoing medical and physiotherapeutic assessments. Approval to conduct the study was obtained from the Bioethics Committee at the Regional Medical Chamber in Krakow (164/KBL/OIL/2021). The study was also registered with the Australian New Zealand Clinical Trials Registry (ACTRN12622000734763). Each volunteer received comprehensive information about the study. If any questions arose, participants had the opportunity to seek clarification before providing written informed consent to participate.

### 2.1 Participant characteristics

The study included 30 participants (17 women and 13 men). Following enrollment, participants were randomly assigned to one of three intervention groups using a simple randomization procedure with sealed, opaque envelopes. No stratification by sex or other baseline characteristics was applied; therefore, the final distribution of women and men within each group resulted solely from the randomization process. The resulting groups comprised:

- Ten participants (six women and four men) performed Vasper Systems LLC training without occlusion or cooling (Group CONT). Age [years]: 23.00 ± 0.00; Body height [cm]: 164.50 ± 6.44; Body mass [kg]: 58.96 ± 10.34.
- Ten participants (five women and five men) performed Vasper Systems LLC training with occlusion (OCC) but without cooling. The pressure in the occlusion cuffs was set at 10 mmHg below the systolic blood pressure (Group OCC). Age [years]: 23.22 ± 0.44; Body height [cm]: 169.83 ± 9.81; Body mass [kg]: 66.31 ± 11.77.
- Ten participants (six women and four men) performed Vasper Systems LLC training with occlusion (OCC) and cooling (COO). Occlusion was applied as described for the OCC group and was additionally combined with activation of the cooling system using cooling cuffs and cooling mats located under the feet and the seat (Group OCC-COO). Age [years]: 23.25 ± 0.46; Body height [cm]: 172.13 ± 9.07; Body mass [kg]: 69.45 ± 13.99.

Inclusion criteria included age 20–25 years, good general health, no comorbidities, no contraindications to HIIT, no regular participation in physical training, and no changes in diet before or during the study (dietary information was self-reported and was not monitored during the study).

All participants had moderately active lifestyles, occupations of similar physical demands, and predominantly sedentary daily routines. Participants reported a daily water intake of 2 L and adherence to a healthy, balanced diet (Mediterranean or Dietary Approaches to Stop Hypertension [DASH]); however, these data were obtained during interviews and were not independently monitored. Physical activity levels, occupational activity, and dietary habits were assessed based on self-reports obtained during the recruitment process. None of the participants had previously participated in this form of training.

### 2.2 Analysis of blood parameters

Blood samples were collected from fasting participants via the basilic, cephalic or median vein on six occasions (2 weeks before the start of training, on the day of training, the morning after the first training, the morning after the 10th training, the morning after the 20th training, and 2 weeks after the end of training) using 6-mL vacuum tubes containing a coagulation activator for serum analysis. Sample collection was performed by a qualified laboratory diagnostician in accordance with established protocols. Blood samples were always collected in the morning (06:00–07:00) after an overnight fast. Serum concentrations of TC, non-HDL, HDL, LDL, and TG were determined using colorimetric assays (Abbott Laboratories, Texas, USA). Analyses were performed using the Alinity C analyzer (Abbott Laboratories, Texas, USA).

### 2.3 Description of the intervention

Two weeks before the first measurement, each participant underwent an exercise test with blood lactate assessment (Żołądź Test on the Vasper device) to determine an individualized training workload below the lactate threshold. The mean resting blood lactate concentration was 0.82 mmol/l (SD: 0.32 mmol/l), and the mean concentration during the exercise included in the analysis was 3.42 mmol/l (SD: 1.10 mmol/l). After the test, 14 participants trained at Vasper load level 9, seven participants at level 11, seven participants at level 13, and two participants at level 15. This workload was established before the first training session and remained unchanged throughout the entire 7-week intervention. No adjustments to the training load were made during the study.

Each participant took part in 20 training sessions (every other day, Monday–Wednesday–Friday, over a 7-week period). The training took place at the Functional Diagnostics Laboratory of the University of Physical Culture in Krakow.

- OCC-COO group: HIIT combined with occlusion (applied to the arms and thighs) and local cooling (using a built-in cryotherapy system beneath the feet and the seat). Each session lasted approximately 24 min and consisted of:

  • Introductory phase (approximately 2 min): warm-up and preparation for exercise

  • Main phase (approximately 20 min): six 3-minute exercise intervals separated by 1-minute rest periods

  • Final phase (approximately 2 min): cool down and conscious muscle relaxation

- OCC group: The training protocol was identical to that of the OCC-COO group without cooling.
- CONT group: The training protocol was identical to that of the other groups but without occlusion or cooling.

### 2.4 Statistical analysis

Descriptive statistics, including the mean (x) and standard deviation (SD), were calculated. The normality of distributions was verified with the Shapiro–Wilk test. Comparisons within and between groups were performed using repeated-measures ANOVA. For between-group comparisons, multivariate ANOVA was used. If significant effects were observed, *post hoc* tests were performed. A significance level of p <0.05 was adopted for all analyses. To determine the sample size, the formula for the minimum required sample size was used, assuming a 95% confidence interval, a population proportion of 0.5, and a maximum error of 5%. The analyses were performed using Statistica 13 software (Tibco Software Inc., USA).

## 3 Results

Analysis of the results showed changes in the mean HDL values in the OCC-COO (F = 3.6868, p = 0.007744, Eta-squared = 0.315467) and OCC (F = 3.1597, p = 0.020789, Eta-squared = 0.344959) groups. Training in both groups resulted in a decrease in HDL levels, followed by stabilization. Differences in the mean HDL values between the OCC-COO and OCC groups were also observed at assessments II, IV, V, and VI (**Tables 1**–**4**).

**Table 1:** Tested indicators (mean ± standard deviation).

Parameters	Group	I–2 weeks before the start of training	II—on the day of training	III—after the first training (next morning)	IV—after the 10th training (next morning)	V—after the 20th training (next morning)	VI—2 weeks after the end of training
TC [mg/dl]	OCC-COO	195.68 ± 44.18	184.59 ± 39.22	182.80 ± 35.38	176.00 ± 40.71	185.16 ± 32.46	195.58 ± 36.17
	OCC	184.46 ± 37.34	191.94 ± 30.68	185.16 ± 29.65	188.37 ± 36.80	190.99 ± 40.51	186.64 ± 35.67
	CONT	183.55 ± 37.20	184.92 ± 30.35	172.20 ± 17.85	166.93 ± 14.24	184.90 ± 36.22	181.40 ± 27.69
non-HDL [mg/dl]	OCC-COO	139.74 ± 46.78	128.56 ± 46.00	128.59 ± 44.49	124.06 ± 46.36	136.01 ± 35.89	144.60 ± 40.88
	OCC	119.26 ± 32.95	125.89 ± 29.06	121.99 ± 30.38	124.70 ± 37.90	130.11 ± 37.22	125.54 ± 31.78
	CONT	125.93 ± 39.44	130.20 ± 31.94	116.30 ± 16.93	113.42 ± 12.23	129.65 ± 31.02	123.23 ± 25.50
HDL [mg/dl]	OCC-COO	55.93 ± 9.58	56.03 ± 10.88	54.21 ± 10.72	51.94 ± 9.43	49.14 ± 11.11	50.98 ± 8.57
	OCC	65.20 ± 7.44	66.05 ± 6.63	63.18 ± 11.10	63.67 ± 6.24	60.88 ± 7.88	61.10 ± 6.08
	CONT	57.62 ± 12.10	54.72 ± 13.51	55.90 ± 10.74	53.52 ± 13.71	55.25 ± 15.90	58.17 ± 15.95
LDL [mg/dl]	OCC-COO	122.15 ± 45.49	114.06 ± 44.88	111.40 ± 38.07	107.93 ± 44.39	119.14 ± 34.20	122.43 ± 34.51
	OCC	107.74 ± 33.92	112.92 ± 27.34	109.19 ± 29.77	110.35 ± 32.24	116.35 ± 36.41	109.89 ± 31.83
	CONT	111.17 ± 34.50	116.58 ± 30.14	103.58 ± 16.15	97.49 ± 11.34	115.48 ± 30.27	107.48 ± 23.35
TG [mg/dl]	OCC-COO	96.83 ± 33.46	77.48 ± 22.99	90.70 ± 48.99	85.92 ± 38.11	91.56 ± 47.92	121.09 ± 65.34
	OCC	60.74 ± 16.66	69.59 ± 17.56	67.99 ± 14.93	76.37 ± 41.94	74.23 ± 20.10	85.61 ± 14.51
	CONT	77.42 ± 39.65	72.52 ± 22.08	65.95 ± 11.99	85.37 ± 37.27	76.03 ± 24.58	85.13 ± 33.18

**Table 2:** Analysis of variance for repeated measurements for the studied indicators in groups - F test value and significance level p.

Parameters	Groups	F test value	Significance level p	Eta-squared
TC [mg/dl]	OCC-COO	1.6493	0.169347	0.170922
	OCC	0.0612	0.997258	0.010104
	CONT	0.9663	0.457163	0.161953
non-HDL [mg/dl]	OCC-COO	1.95987	0.105801	0.196776
	OCC	0.0577	0.997622	0.009522
	CONT	0.8758	0.511419	0.149053
HDL [mg/dl]	OCC-COO	3.6868	0.007744 *	0.315467
	OCC	3.1597	0.020789 *	0.344959
	CONT	1.1250	0.372844	0.183676
LDL [mg/dl]	OCC-COO	1.15158	0.349680	0.125834
	OCC	0.0434	0.998800	0.007176
	CONT	1.0536	0.409027	0.174050
TG [mg/dl]	OCC-COO	1.88745	0.118140	0.190893
	OCC	1.1580	0.352490	0.161777
	CONT	0.95177	0.465550	0.159914

*significance level of p = 0.05.

**Table 3:** *Post hoc* test values for the studied indicators depending on the study.

Parameters	Study	I–2 weeks before the start of training	II—on the day of training	III—after the first training (next morning)	IV—after the 10th training (next morning)	V—after the 20th training (next morning)	VI—2 weeks after the end of training
HDL [mg/dl] OCC-COO	I		0.961631	0.409395	0.060594	0.002117 *	0.021194 *
	II	0.961631		0.382983	0.054683	0.001849 *	0.018878 *
	III	0.409395	0.382983		0.279085	0.018635 *	0.125408
	IV	0.060594	0.054683	0.279085		0.182878	0.642358
	V	0.002117 *	0.001849 *	0.018635 *	0.182878		0.380116
	VI	0.021194 *	0.018878 *	0.125408	0.642358	0.380116	
HDL [mg/dl] OCC	I		0.532039	0.541238	0.453144	0.032826 *	0.060147
	II	0.532039		0.988759	0.174073	0.007457 *	0.014821 *
	III	0.541238	0.988759		0.178373	0.007723 *	0.015326 *
	IV	0.453144	0.174073	0.178373		0.149958	0.242072
	V	0.032826 *	0.007457 *	0.007723 *	0.149958		0.778256
	VI	0.060147	0.014821 *	0.015326	0.242072	0.778256	

*significance level of p = 0.05.

**Table 4:** Intergroup comparison for HDL.

Parameters	Study	Mean (OCC-COO)	Mean (OCC)	Mean (CONT)	p OCC-COO/CONT	p OCC/CONT	p OCC-COO/OCC
HDL [mg/dl]	I	55.93	65.20	57.62	0.768275	0.193206	0.053613
	II	56.03	66.05	54.72	0.838829	0.059463	0.039666 *
	III	54.21	63.18	55.90	0.769933	0.242118	0.111191
	IV	51.94	63.67	53.52	0.795389	0.104908	0.013278 *
	V	49.14	60.88	55.25	0.395632	0.398593	0.025511 *
	VI	50.98	61.10	58.17	0.275697	0.639249	0.014197 *

*significance level of p = 0.05.

## 4 Discussion

The findings of studies investigating the effects of various forms of physical training on blood lipid profiles remain inconsistent and, at times, inconclusive. These discrepancies are largely attributable to substantial methodological heterogeneity across studies. Key contributing factors include differences in participant characteristics, such as age, baseline physical fitness, and health status. In addition, training protocols vary considerably with respect to exercise modality, intensity, frequency, and intervention duration. A notable limitation of many studies is the lack of adequate dietary control, despite diet being a critical determinant of lipid metabolism. Furthermore, environmental influences—such as seasonal variation—are frequently overlooked, despite their potential to significantly modulate lipid profiles. Consequently, direct comparison of findings across studies is challenging, limiting the formulation of clear, generalizable conclusions regarding the relationship between exercise modalities and changes in biochemical and biophysical parameters.

The present study investigated the effects of high-intensity interval training combined with blood flow restriction, with or without local cooling, on lipid profile parameters in healthy young adults. The principal finding was that repeated exposure to HIIT combined with blood flow restriction, irrespective of cooling, was associated with a decrease in HDL-C concentrations, whereas no significant changes were observed in total cholesterol, LDL-C, non-HDL cholesterol, or triglycerides. In contrast, the control group performing HIIT without additional interventions showed no unfavorable changes in the lipid profile. Importantly, the observed reduction in HDL-C occurred after repeated training sessions and was not evident after a single exercise bout.

Most previous studies investigating conventional HIIT have reported either improvements or no significant changes in the lipid profile. In a systematic review and meta-analysis, Wood et al. (2019) concluded that HIIT was not superior to moderate-intensity continuous training in modifying total cholesterol, LDL-C, triglycerides, or the TC/HDL ratio, although HIIT appeared to promote greater increases in HDL-C. Similarly, Lira et al. (2019) demonstrated that a five-week HIIT intervention induced favorable changes in several cardiometabolic markers and a tendency toward increased HDL-C concentrations. In individuals with obesity and diabetes, Zhu et al. (2024) observed improvements in HDL function and lipid metabolism following short-term HIIT, although responses differed according to metabolic status. Therefore, the reduction in HDL-C observed in the present study appears to contrast with most findings reported for conventional interval training.

However, it should be emphasized that the present intervention differed substantially from conventional HIIT protocols because exercise was combined with blood flow restriction and, in one group, additional local cooling. Consequently, the observed responses cannot be attributed to HIIT alone. Blood flow restriction training produces substantial metabolic stress and may augment inflammatory and muscle damage responses associated with intense exercise (Sarkar et al., 2021; Leite et al., 2023). Furthermore, repeated high-intensity efforts have been linked to calcium accumulation, oxidative stress, and chronic low-grade inflammation, potentially affecting metabolic adaptations (Kano et al., 2012). Therefore, it is possible that the addition of blood flow restriction and cooling altered the physiological response to interval exercise and modified lipoprotein metabolism differently than traditional HIIT protocols.

Another explanation may be related to the duration of the intervention. Previous studies suggest that favorable adaptations in lipid metabolism become more pronounced after longer interventions. Racil et al. (2016) and Khammassi et al. (2018) reported improvements in the lipid profile after training programs lasting 8–12 weeks. Therefore, the seven-week intervention applied in the present study may have been insufficient to induce beneficial adaptations in HDL metabolism. Moreover, all participants had normal baseline lipid values, which may have limited the magnitude of exercise-induced improvements. It has previously been suggested that individuals with more favorable baseline lipid profiles exhibit smaller responses to training interventions (Gordon et al., 2014).

Sex-related differences may also partly explain the discrepancies between our findings and previous reports. Exercise-induced changes in HDL-C appear to differ between men and women (Wilmore, 2001). Berglund et al. (2021) demonstrated sex-specific responses to long-term HIIT, with beneficial effects on HDL-C observed primarily in men. Although the distribution of men and women across the groups was relatively balanced, sex-specific analyses were not performed because of the limited sample size. Consequently, a potential influence of sex on HDL-C responses cannot be excluded and should be investigated in future studies involving larger cohorts.

Another important consideration is that HDL-C concentration alone does not fully reflect HDL functionality. Recent evidence indicates that exercise-induced cardioprotective effects may be mediated by improvements in HDL quality rather than by increases in HDL-C concentration itself. Zhu et al. (2024) demonstrated that HIIT enhanced cholesterol efflux capacity and antioxidant properties of HDL particles despite heterogeneous changes in HDL-C concentration. Similarly, Stanton et al. (2022) reported improvements in HDL function and the lipoprotein profile following exercise interventions. Therefore, the decrease in HDL-C observed in the present study does not necessarily indicate impaired antiatherogenic function, and future investigations should include assessments of HDL functionality, apolipoproteins, and cholesterol efflux capacity to better characterize exercise-induced adaptations.

From a physiological perspective, the combination of HIIT with BFR and local cooling may induce adaptations that differ from those elicited by conventional HIIT alone. BFR increases metabolic stress by reducing oxygen availability and promoting metabolite accumulation within the working muscles, thereby enhancing anaerobic metabolism and the activation of hypoxia-related signaling pathways. Repeated exposure to these conditions may also augment oxidative stress and transient inflammatory responses, both of which have been implicated in alterations of lipoprotein metabolism (Kano et al., 2012; Pearson and Hussain, 2015; Boeno et al., 2018; Patterson et al., 2019; Sarkar et al., 2021; Leite et al., 2023). In addition, local cooling induces peripheral vasoconstriction and modifies tissue perfusion, potentially affecting substrate utilization, endothelial function, and post-exercise recovery (Anz et al., 2018; Callanan et al., 2021). The simultaneous application of BFR and local cooling may create a distinct physiological environment characterized by altered tissue perfusion, metabolic stress, and recovery processes, which may influence lipid metabolism differently from conventional HIIT. Furthermore, recent evidence suggests that exercise-induced changes in HDL-C concentration do not necessarily reflect alterations in HDL functionality, as improvements in cholesterol efflux capacity and antioxidant properties may occur despite unchanged or even reduced HDL-C concentrations (Spranger et al., 2015; Stanton et al., 2022; Zhu et al., 2024). Although these mechanisms provide biologically plausible explanations for the observed reduction in HDL-C, they remain speculative because inflammatory markers, oxidative stress biomarkers, endothelial function, and HDL functionality were not assessed in the present study.

The discrepancies between the present findings and previous studies may also result from methodological differences, including participant characteristics, exercise protocols, intervention duration, and nutritional factors. Dietary intake and habitual physical activity outside the intervention were assessed only by self-report and were not objectively monitored. Since nutritional habits represent one of the strongest determinants of lipid metabolism (Gordon et al., 2014), their contribution to the observed HDL-C changes cannot be excluded. Moreover, inflammatory markers, oxidative stress biomarkers, and indices of muscle damage were not assessed, limiting mechanistic interpretation of the results.

Several limitations should be acknowledged. First, the relatively small sample size reduced statistical power and precluded sex-specific analyses. Second, the intervention lasted only seven weeks and included young, healthy individuals with normal baseline lipid values, which restricts the generalizability of the findings. Third, dietary intake and physical activity outside the intervention were not objectively monitored. Finally, HDL functionality, inflammatory markers, apolipoproteins, and oxidative stress parameters were not assessed, preventing identification of the mechanisms underlying the observed decrease in HDL-C.

In conclusion, HIIT combined with blood flow restriction, with or without local cooling, was associated with a transient reduction in HDL-C concentrations in healthy young adults, whereas no changes were observed in other lipid parameters. Given that HDL-C values remained within physiological ranges and HDL functionality was not assessed, the clinical significance of these findings remains uncertain. Future studies involving larger populations, longer intervention periods, and comprehensive assessments of lipoprotein function and inflammatory responses are needed to clarify the mechanisms underlying these observations.

## 5 Conclusions

HIIT combined with blood flow restriction (BFR)and cooling, as well as BFR alone, may negatively affect HDL-C levels in young, healthy individuals. In contrast, regular submaximal interval exercise performed without additional modifications appears to be safe and does not adversely affect the lipid profile. Importantly, changes in HDL-C levels occur following repeated training sessions rather than after a single exercise bout. Since HDL-C values remained within physiological ranges and no changes were observed in the other lipid parameters, the clinical significance of this finding remains uncertain.

This study is subject to several limitations. These include the relatively small sample size, the restriction of the study population to young adults, and the inclusion of participants with only low to moderate levels of physical activity. Consequently, caution is warranted when generalizing these findings to broader populations. Furthermore, the relatively short duration of the intervention may not adequately reflect long-term effects. Future studies should involve larger, more heterogeneous populations and investigate the long-term effects of HIIT combined with blood flow restriction and localized cryotherapy.

## Data Availability

The original contributions presented in the study are included in the article/supplementary material. Further inquiries can be directed to the corresponding author.
